# Effectiveness of Context‐Specific Social and Behaviour Change (SBC) Interventions in Improving Child Diet in Ethiopia: A Systematic Review and Meta‐Analysis

**DOI:** 10.1111/mcn.70245

**Published:** 2026-09-06

**Authors:** Taddese Alemu Zerfu, Amare Abera Tareke, Meron Girma, Tirsit Genye, Bedassa Tessema, Frezer Zewdu, Melaku Bayable, Archana Sarkar, Masresha Tessema, Aregash Samuel Hafebo

**Affiliations:** ^1^ International Food Policy Research Institute (IFPRI) Addis Ababa Ethiopia; ^2^ College of Medicine and Health Sciences Wollo University Dessie Ethiopia; ^3^ Ethiopian Public Health Institute Addis Ababa Ethiopia; ^4^ Federal Ministry of Health Nutrition Coordination Office Addis Ababa Ethiopia; ^5^ Federal Ministry of Education Addis Ababa Ethiopia; ^6^ Deutsche Gesellschaft für Internationale Zusammenarbeit (GIZ) Bonn Germany; ^7^ Deutsche Gesellschaft für Internationale Zusammenarbeit (GIZ) Addis Ababa Ethiopia

**Keywords:** child nutrition, dietary diversity, Ethiopia, SBC interventions, weight

## Abstract

In Ethiopia, malnutrition reflects stark nutritional disparities across residences and regions, with young children in rural areas exhibiting markedly poorer outcomes than their urban counterparts. This systematic review and meta‐analysis evaluated the effectiveness of context‐specific social and behaviour change (SBC) interventions, including nutrition education, on child nutrition, measured through changes in Minimum Dietary Diversity (MDD), Dietary Diversity Score (DDS), and child weight gain. We systematically searched PubMed, Scopus, HINARI, and Google Scholar for studies on SBC interventions in Ethiopia. After screening for eligibility, data were extracted on MDD, DDS, and weight gain. Using random‐effects models, pooled risk ratios (RR) and mean differences (MD) were calculated, heterogeneity was assessed, and subgroup analyses were conducted by region to examine variations in effectiveness. A total of 33 studies met the inclusion criteria. Overall, SBC interventions were associated with improvements in child dietary diversity and weight outcomes. Children in intervention groups were 67% more likely to meet MDD (RR = 1.67; 95% CI: 1.42–1.96). Dietary diversity scores increased (MD = 0.80; 95% CI: 0.51–1.08), and child weight gain improved (MD = 1.32; 95% CI: 0.49–2.15) compared with controls. Effects were strongest in urban settings, particularly in Addis Ababa. By region, the Southern Nations, Nationalities, and Peoples' Region (SNNPR) showed the highest weight gain (MD = 2.01), while effects in Oromia and Amhara were more moderate and occasionally non‐significant. Heterogeneity was high (*I*² = 99.64%), indicating substantial variability in effectiveness attributable to contextual differences, implementation modalities, and programme intensity across Ethiopia. In Conclusion, SBC interventions significantly improved dietary diversity and child growth outcomes in Ethiopia, although effects varied across regions. Tailoring approaches to local cultural and environmental contexts may achieve stronger impacts. Integrating SBC with initiatives that strengthen food security, enhance health service delivery, and address structural barriers is essential for maximizing effectiveness.

## Summary

- A systematic review and meta‐analysis of 33 studies found that context‐specific social and behaviour change interventions improved child dietary outcomes in Ethiopia.
- SBC interventions increased the likelihood of achieving minimum dietary diversity by 67% and also improved dietary diversity scores and child weight outcomes.
- Effects varied substantially across regions and study contexts, highlighting the importance of adapting interventions to local food environments, cultural practices, household circumstances, and implementation conditions.
- Policies and programmes should integrate context‐specific SBC approaches with broader efforts to address food security, health services, and structural barriers to improve child nutrition.

## Graphical abstract

This systematic review and meta‐analysis shows that SBC interventions are associated with improved child dietary diversity and nutrition outcomes in Ethiopia. However, effectiveness varies across regions, highlighting the importance of context‐specific strategies and integrated approaches to strengthen child nutrition.

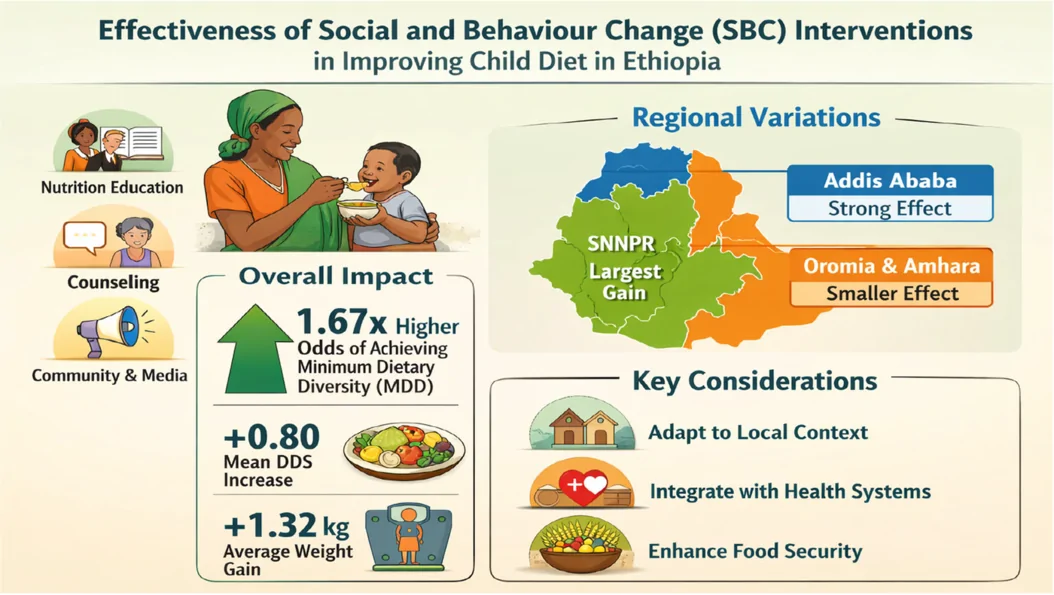

## 1 Introduction

Malnutrition remains a major public health challenge in many low‐ and middle‐income countries (LMICs), with infants, children, women, and other socially and economically vulnerable populations disproportionately affected (WHO & UNICEF 2023; World Health Organization 2024). In Ethiopia, malnutrition is characterised by persistent undernutrition, widespread micronutrient deficiencies, and a growing prevalence of overweight and obesity. Together, these overlapping forms of malnutrition constitute the triple burden of malnutrition, reflecting the country's ongoing nutrition transition and the increasing complexity of its public health challenges. While undernutrition and micronutrient deficiencies remain widespread, overweight and obesity are rising rapidly, particularly in urban areas, further complicating efforts to improve population nutrition and health (WHO & UNICEF 2023; EPHI & ICF 2021; FMoH, Ethiopia 2023).

Addressing the triple burden of malnutrition in Ethiopia requires more than simply increasing food availability (Sebsbie et al. 2022; Yigezu et al. 2024); it requires addressing the social, economic, and behavioural factors that shape food access, food choice, and dietary practices (FAO, IFAD, UNICEF, WFP, & WHO 2023). Evidence from Ethiopia shows that household socioeconomic conditions, maternal education, women's empowerment, nutrition knowledge, and food environments are important determinants of dietary diversity (Woldegebriel et al. 2020; Baye et al. 2024; Kuche et al. 2020). A transformative response should therefore integrate food‐based and nutrition‐sensitive interventions with community nutrition education and gender‐responsive approaches, rather than treating food production or availability as sufficient conditions for improved diet (Bliznashka et al. 2022; MoH 2018; Margolies et al. 2022).

Dietary diversity is a critical measure of diet quality, directly linked to adequate micronutrient intake, healthy growth, and overall well‐being, particularly among children and women (World Health Organization & United Nations Children's Fund 2021). When diets lack variety, the risks escalate: nutrient deficiencies weaken immunity, while long‐term developmental delays undermine human potential. Despite Ethiopia's concerted efforts to improve nutrition through targeted programmes, dietary diversity remains alarmingly low nationwide (Bitew et al. 2021; Jateno et al. 2023). Recent data paints a stark picture: just 14% of children aged 6–23 months meet the minimum dietary diversity standard, with rates plunging even lower in Oromia and other emerging regions like Afar, Somali (EPHI & ICF 2021). This gap perpetuates a vicious cycle of malnutrition—fuelling micronutrient deficiencies, stunting, and wasting—with consequences that ripple across generations.

Social and Behaviour Change Communication (SBC) can influence dietary behaviours and nutrition outcomes by addressing the social norms, beliefs, knowledge, and practices that shape food choices and feeding behaviours (Mahumud et al. 2022; Workicho et al. 2021; Litvin et al. 2024). Using mass media, community activism, interpersonal counselling, advice sessions and expert representatives from the community, SBC raises public consciousness of healthy diets and skills for food preparation (Girard et al. 2021; Thapliyal et al. 2024). However, the effectiveness of these interventions is highly context‐dependent; in a culturally diverse country such as Ethiopia, SBC strategies must be carefully tailored to local contexts to ensure they are both relevant and attended to by target populations.

Ethiopia's regions are characterised by widely differing cultural practices, livelihood systems, dietary habits, and food security levels. Understanding how context‐specific SBC interventions influence dietary diversity is essential for tailoring nutrition programmes and achieving sustainable benefits.

Although several studies have explored the impact of SBC interventions on nutrition outcomes in Ethiopia (Abuye et al. 2024; Han et al. 2021; Kang et al. 2017a; Kim et al. 2019), the findings remain fragmented and inconsistent, with limited focus on dietary diversity as a key outcome. Variability in design, implementation, and delivery of SBC interventions has led to inconsistent findings on their impact on dietary diversity. A systematic review and meta‐analysis are needed to consolidate evidence, assess effectiveness, and inform more targeted, region‐specific SBC strategies in Ethiopia. Therefore, this study aimed to synthesise existing evidence from both published and grey literature to assess the effectiveness of region‐specific SBC interventions on improving dietary diversity in Ethiopia.

## 2 Methods

### 2.1 Literature Search

A comprehensive literature search was conducted to identify studies assessing the effectiveness of SBC interventions on dietary diversity in Ethiopia. The search was performed in four electronic databases: PubMed, Scopus, Hinari, and Google Scholar.

For the purposes of this review, we adopted a clear operational definition of SBC interventions: any intervention with explicit behaviour‐change objectives delivered through interpersonal and/or media‐based communication channels. SBC interventions (including nutrition education) were included when embedded within this framework, specifically, when it involved structured opportunities for dialogue, problem‐solving, or skills‐building aimed at influencing dietary practices, rather than passive information transfer alone. To support comparability across studies and to facilitate interpretation of heterogeneity, we developed a simple coding framework to classify SBC components. This framework categorised interventions according to delivery strategy (e.g., interpersonal counselling, community mobilisation, mass media, digital platforms, or integration with other services). Throughout this review, we consistently refer to these interventions as ‘SBC (including nutrition education)’ in the Methods and inclusion criteria to reflect the scope of our synthesis accurately.

The following search terms and combinations were used, with the full Boolean strings for each database provided in Additional Table 1: nutrition education, nutrition counselling, nutrition intervention, nutrition promotion, nutrition literacy, along with keywords and MeSH terms including (nutri, diet, feed, food, vitamin*, nutritional status, nutritive value, diet, healthy eating, infant nutrition disorders, nutritional requirements, malnutrition, dietary diversity, diet diversity, dietary variety, food variety, dietary diversity score, minimum dietary diversity, diet, food, and nutrition).

The search was further refined by including the country name ‘Ethiopia’ and restricting results to primary studies. To filter out non‐empirical research, we applied Boolean operators to exclude records with the terms ‘Systematic Review’, ‘Meta‐analysis’, or ‘Scoping Review’ in the title. For Google Scholar, the search was limited to the title field to improve specificity, yielding 122 records. All records were imported into Rayyan and de‐duplicated prior to screening.

No date restrictions were applied to ensure comprehensive coverage. The search was limited to English‐language studies; however, as interventional research in Ethiopia is overwhelmingly published in English, the risk of language bias is minimal. The last search was conducted on July 15, 2024. This review was conducted and reported in accordance with the Preferred Reporting Items for Systematic Reviews and Meta‐Analyses (PRISMA) guidelines (Page et al. 2021).

### 2.2 Inclusion and Exclusion Criteria

We included randomised controlled trials (RCTs), quasi‐experimental studies and adequacy evaluations with an intervention and comparison group, where SBC interventions, including nutrition education and counselling, were a central component of the package delivered. Studies were eligible if they reported on dietary diversity (or dietary diversity score) as an outcome. Because this review originated from a broader registered protocol evaluating nutrition‐related outcomes across multiple population groups, studies involving different target populations were initially eligible. However, for the present review, only child dietary outcomes (minimum dietary diversity, dietary diversity score and child weight) were synthesised in the primary analyses. Studies involving other populations contributed only where they reported child outcomes separately or were included in predefined subgroup analyses. Both published and unpublished studies written in English were considered.

We excluded observational studies without a comparison group, as well as systematic reviews, meta‐analyses and scoping reviews (filtered during the search process). Additional exclusion criteria included opinion‐based papers, conference abstracts and studies for which the full text was unavailable. For studies where SBC interventions (including nutrition education) were combined with other interventions (e.g., vouchers, agriculture programmes), we only included those that isolated and reported the effect of the SBC component compared to a non‐SBC control group. Although we initially planned to include both qualitative and quantitative primary research, only quantitative studies were identified.

### 2.3 Screening

The search results were exported to Rayyan, an open‐source software for article screening. Duplicates were removed and screening was performed. A two‐stage study screening process was performed. In the first stage, the search results were screened based on the title and abstract by two reviewers. In the second stage, after the initial screening, the same two reviewers performed a full‐text screening. Any disagreements that arose during each stage were resolved through consensus.

### 2.4 Data Extraction

A standard data extraction tool containing relevant study characteristics, intervention status, study outcomes and possible sources of heterogeneity was developed in accordance with the Cochrane Collaboration and the Centre for Review and Dissemination. The tool extracted study details such as author, publication year, study design, region, population and SBC interventions (including nutrition education), as well as main findings, both qualitative and quantitative. During data extraction, a single study may report various nutrition outcomes; we extracted these outcomes separately for analysis. Two independent authors extracted the data. Differences during extraction were resolved by discussion and consensus.

### 2.5 Intervention

The intervention of this study is SBC interventions (including nutrition education). Although some interventions targeted mothers, caregivers, adolescents or households, outcomes included in this review were restricted to child nutrition indicators.

### 2.6 Outcome

The primary outcome of this meta‐analysis was child dietary diversity among children aged 6–23 months whenever reported, measured as dietary diversity score, minimum dietary diversity, or adequate dietary diversity, as reported in the original studies. Included studies were required to assess the effect of nutrition education on dietary diversity using a quantifiable effect size.

### 2.7 Quality Assessment

Risk of bias for included RCTs was judged in accordance with the Cochrane Collaboration Risk of Bias Tool 2 (ROB) (Sterne et al. 2019), for reporting of sequence generation, allocation concealment, use of blinding of participants and personnel, loss to follow up and other biases. The methodological quality of quasi‐experimental studies were judged by Risk Of Bias In Non‐Randomised Studies ‐ of Interventions (ROBINs‐I) (Sterne et al. 2016).

Dietary diversity indicators were analysed separately according to the population‐specific indicator used in each study. Child dietary diversity scores were never pooled with women's dietary diversity scores because these indicators are based on different food group classifications. Where studies involving other populations were included, they were analysed only within separate subgroup analyses and were not combined with child DDS estimates.

### 2.8 Data Analysis

The data analysis approach was informed by the reporting patterns and methodological heterogeneity of the included studies. All statistical analyses were conducted using STATA software (Version 17, StataCorp, College Station, TX, USA). The analysis focused on assessing the effect of SBC interventions (including nutrition education) on dietary diversity, which is expected to be measured using various outcome indicators (e.g., dietary diversity score, minimum dietary diversity, adequate dietary diversity). Given the expected variability in study populations, intervention contexts, and outcome definitions, heterogeneity was anticipated.

To address this, we stratified analyses based on key variables such as population group, type and duration of the intervention, and geographic setting—subject to the availability of a sufficient number of studies within each stratum. Data were extracted and summarised using structured tables, and findings from the systematic review were presented both narratively and in tabular form.

Where adequate comparable data existed, nutrition outcomes were pooled for meta‐analysis. Given the likelihood of multiple outcomes being reported within a single study, efforts were made to extract or compute standardised effect sizes where possible. All pooled risk ratios (RR) were calculated from raw event counts reported in the original studies and therefore represent unadjusted effect estimates. All pooled estimates were reported with 95% confidence intervals (CIs), using a random‐effects model based on the inverse variance method to account for between‐study variability.

Statistical heterogeneity was assessed using the *I* ^2^ statistic, with *I* ^2^ values of approximately 25%, 50%, and 75% interpreted as low, moderate, and high heterogeneity, respectively (Higgins and Thompson 2002) Where sufficient data allowed, subgroup analyses were conducted to explore potential sources of heterogeneity.

We performed several sensitivity analyses to assess the robustness of our pooled estimates. First, we used a leave‐one‐out approach, sequentially removing individual studies to examine their influence on the overall effect size. Second, to evaluate whether the inclusion of weaker study designs biased the results, we conducted a sensitivity analysis restricted to RCTs only. Third, we stratified studies by design (RCTs vs. quasi‐experimental studies) and by risk of bias rating in subgroup analyses. To assess potential publication bias and small‐study effects, we visually inspected funnel plots and performed Egger's regression test. A *p*‐value of ≤ 0.05 was considered statistically significant for all analyses.

## 2.9 | Ethics Statement

This systematic review and meta‐analysis used only data extracted from previously published and publicly available studies and did not involve the collection of primary human participant data. Therefore, institutional ethical approval was not required.

## 3 Results

### 3.1 Protocol Registration

The protocol of this study is prospectively registered on International prospective register of systematic reviews (PROSPERO) with registration number CRD42024571881.

### 3.2 Deviation From the Protocol

The protocol aimed to investigate various nutritional outcomes. From this large pool of nutritional outcomes, this paper presents the findings of three outcomes.

### 3.3 Search Results

**Figure 1:**
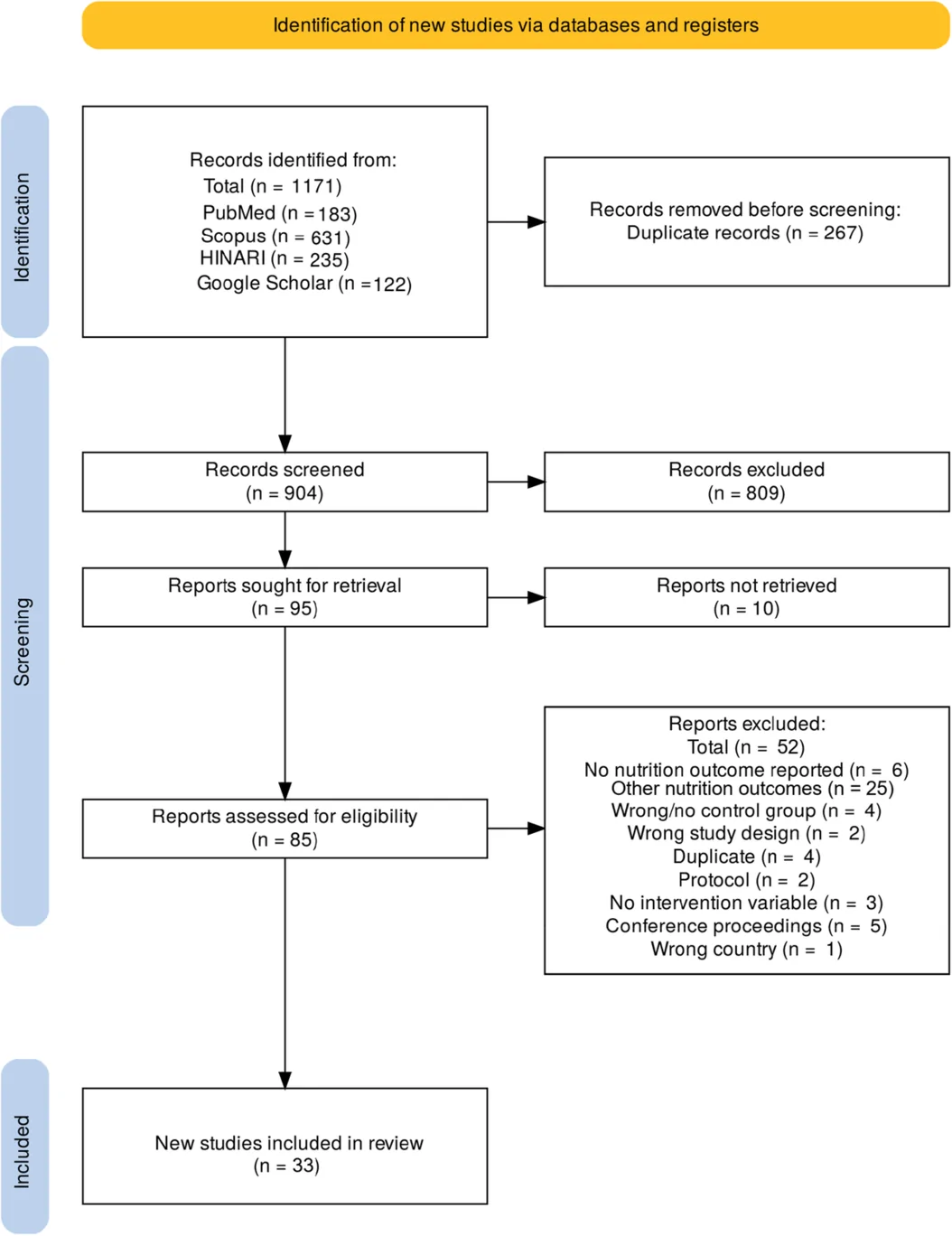
PRISMA flow chart for study screening and selection.

A total of 1171 records were identified through database searches, including PubMed (183), Scopus (631), HINARI (235), and Google Scholar (122). After removing 267 duplicate records, 904 records remained for screening. Following the screening, 809 records were excluded, and 95 reports were sought for retrieval. Of these, 10 reports could not be retrieved, leaving 85 reports assessed for eligibility. Ultimately, 52 reports were excluded for reasons such as no nutrition outcome reported (*n* = 6), other nutrition outcomes (*n* = 25), wrong or no control group (*n* = 4), wrong study design (*n* = 2), duplicates (*n* = 4), protocols (*n* = 2), no intervention variable (*n* = 3), conference proceedings (*n* = 5), and wrong country (*n* = 1). Finally, 33 distinct studies (Workicho et al. 2021; Gebremichael and Lema 2024; Wakwoya et al. 2023; Tesfaye et al. 2024; Bidira et al. 2022; Beressa et al. 2024; Adugna et al. 2024; Tamiru et al. 2017; Kuma et al. 2023; Kang et al. 2017b; Tsegaye et al. 2022; Shuremu et al. 2023; Mekonnen et al. 2023; Seid and Babbel 2023; Mekonnen et al. 2022; Demilew et al. 2020a, 2020; Abiyu and Belachew 2020; Abiyu et al. 2020; Eshete et al. 2023; Teshome et al. 2020; Teshome 2019; Tariku et al. 2015; Muluye et al. 2020; Daba and Ersado 2015; Sisay and Tesfaye 2023; Dansa et al. 2019; Mulualem et al. 2016; Guled et al. 2020; Haileselassie et al. 2022; Kim et al. 2016, 2023; Tessema et al. 2023) were included in the review, Figure 1.

### 3.4 Description of the Included Studies

Characteristics of included studies are presented in the supporting material Additional Table 1. A total of 33 studies evaluating SBC interventions (including nutrition education) interventions across Ethiopia were included in this review. These studies employed either randomised RCTs or quasi‐experimental designs. Target populations ranged widely, including children under 2 years, adolescents, pregnant women, mother child pairs, adults, and elders. Sample sizes varied from 41 (Teshome 2019) to 1796 (Tessema et al. 2023) participants in either intervention or control group, reflecting the heterogeneity of settings and intervention scopes (Workicho et al. 2021; Gebremichael and Lema 2024; Wakwoya et al. 2023; Tesfaye et al. 2024; Bidira et al. 2022; Beressa et al. 2024; Adugna et al. 2024; Tamiru et al. 2017; Kuma et al. 2023; Kang et al. 2017b; Tsegaye et al. 2022; Shuremu et al. 2023; Mekonnen et al. 2023; Seid and Babbel 2023; Mekonnen et al. 2022; Demilew et al. 2020a, 2020; Abiyu and Belachew 2020; Abiyu et al. 2020; Eshete et al. 2023; Teshome et al. 2020; Teshome 2019; Tariku et al. 2015; Muluye et al. 2020; Daba and Ersado 2015; Sisay and Tesfaye 2023; Dansa et al. 2019; Mulualem et al. 2016; Guled et al. 2020; Haileselassie et al. 2022; Kim et al. 2016, 2023; Tessema et al. 2023).

Although the broader review identified studies conducted among women, adolescents, adults, and older people, only studies reporting child dietary outcomes contributed to the pooled child analyses presented below. Results from other population groups are presented only in subgroup analyses designed to explore intervention characteristics.

The interventions were delivered through a variety of contexts. School‐based programmes were used predominantly for adolescents and young children (Bidira et al. 2022; Mekonnen et al. 2023; Tariku et al. 2015; Dansa et al. 2019; Kim et al. 2023), whereas interventions targeting pregnant women, mother child pairs, and elders were frequently implemented through home visits, health institutions, and community‐based programmes.

Intervention content was comprehensive, addressing dietary diversity, complementary feeding practices, meal frequency, healthy eating habits, balanced diets, and nutrition counselling. For children under 2 years, key topics included complementary food preparation, feeding frequency, hygiene and dietary diversification (Muluye et al. 2020; Daba and Ersado 2015; Mulualem et al. 2016; Haileselassie et al. 2022; Kim et al. 2023; Tessema et al. 2023). Adolescents received education on specific food groups, healthy food choices and diet diversity (Mekonnen et al. 2023; Dansa et al. 2019; Kim et al. 2023), while pregnant women and mother child pairs were targeted with interventions emphasising meal frequency, portion size, dietary diversity, birth preparedness and maternal nutrition (Workicho et al. 2021; Gebremichael and Lema 2024; Wakwoya et al. 2023; Tesfaye et al. 2024; Beressa et al. 2024; Adugna et al. 2024; Kuma et al. 2023; Mekonnen et al. 2022; Demilew et al. 2020a, 2020; Abiyu and Belachew 2020; Abiyu et al. 2020; Teshome 2019; Tariku et al. 2015; Daba and Ersado 2015; Sisay and Tesfaye 2023). Adults and elders were primarily exposed to interventions focused on balanced diets, healthy eating habits and lifestyle modification (Shuremu et al. 2023; Seid and Babbel 2023; Eshete et al. 2023).

Modes of intervention delivery were diverse. Direct educational approaches, such as lectures, group discussions and counselling sessions, were frequently combined with interactive methods including role play, peer mentoring, participatory activities and practical demonstrations. Many studies supplemented these approaches with media and printed materials, such as posters, brochures, leaflets, radio messages and SMS communication (Teshome 2019; Muluye et al. 2020; Sisay and Tesfaye 2023; Guled et al. 2020; Kim et al. 2016). Community‐level activities, including food festivals, sensitisation workshops and community dialogues, were used to reinforce learning and foster engagement (Tamiru et al. 2017; Mekonnen et al. 2022; Tessema et al. 2023). The duration of interventions ranged from 2 weeks to 1 year, with most lasting between 3 and 9 months.

Outcomes measured across studies primarily included dietary diversity score (DDS, 12 studies (14 reports)) and minimum dietary diversity (MDD, 19 studies (23 reports), and weight (10 studies (12 reports)). Table summarising the characteristics of included studies is provided in the supporting material [Additional Table 2].

### 3.5 Regional Coverage

**Figure 2:**
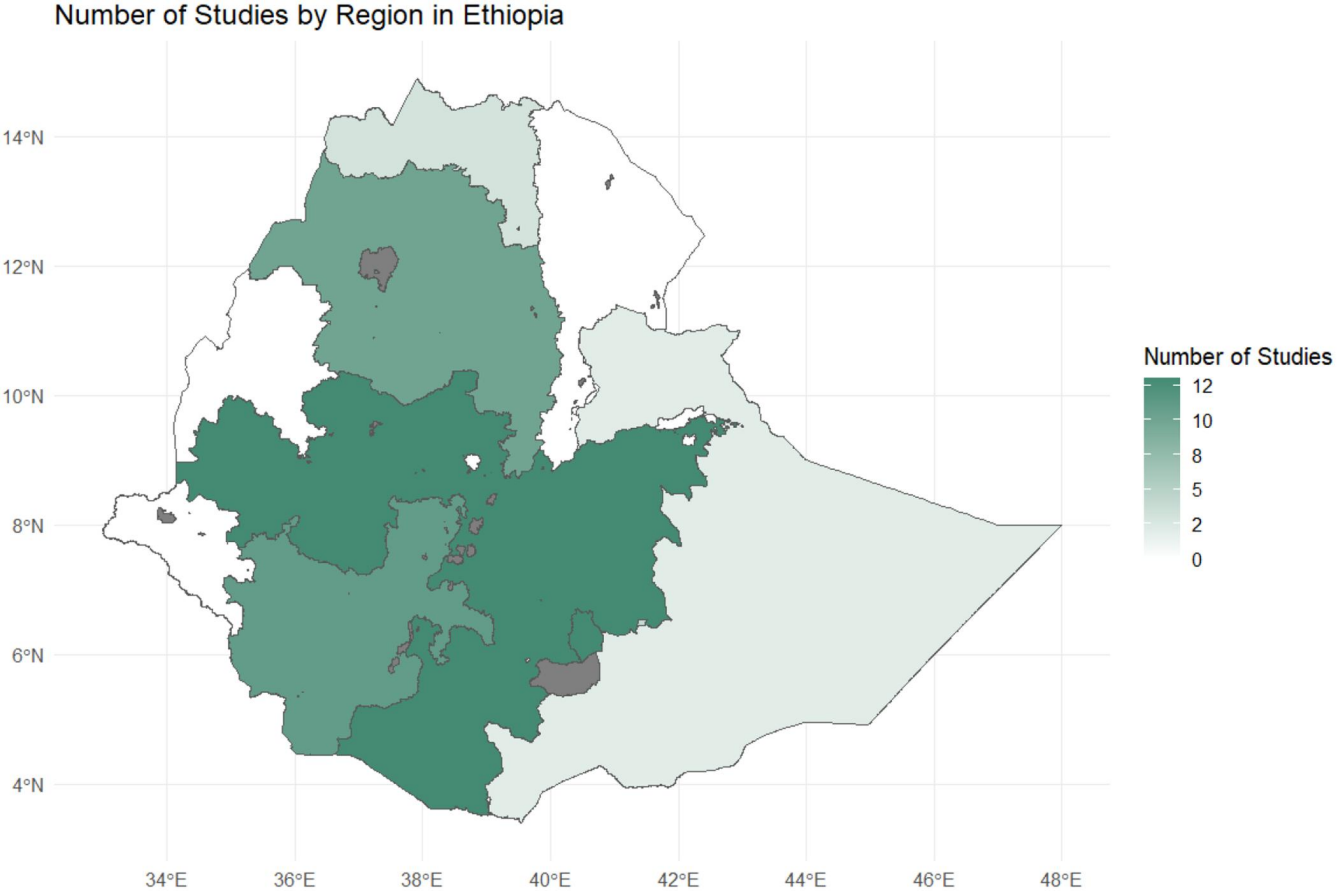
Regional distribution of included studies in Ethiopia. (*Note:* Studies from the newly formed Sidama and Southwest Ethiopia regions are displayed under SNNPR for presentation purposes).

The geographic distribution of studies in the region were from Oromia—13 studies (Workicho et al. 2021; Gebremichael and Lema 2024; Wakwoya et al. 2023; Tesfaye et al. 2024; Bidira et al. 2022; Beressa et al. 2024; Adugna et al. 2024; Tamiru et al. 2017; Kuma et al. 2023; Kang et al. 2017b; Tsegaye et al. 2022; Shuremu et al. 2023; Tessema et al. 2023), South Nations Nationalities and People's Region (SNNPR)—11 studies (Teshome et al. 2020; Teshome 2019; Tariku et al. 2015; Muluye et al. 2020; Daba and Ersado 2015; Sisay and Tesfaye 2023; Dansa et al. 2019; Mulualem et al. 2016; Kim et al. 2016, 2023; Tessema et al. 2023), Amhara—10 studies (Workicho et al. 2021; Mekonnen et al. 2023; Seid and Babbel 2023; Mekonnen et al. 2022; Demilew et al. 2020a, 2020; Abiyu and Belachew 2020; Abiyu et al. 2020; Eshete et al. 2023; Tessema et al. 2023), Tigray—3 studies (Haileselassie et al. 2022; Kim et al. 2016; Tessema et al. 2023), and Somali—2 studies. There are studies conducted in multiple regions, we count them in all regions where they conducted. Figure 2.

### 3.6 Quality of Included Studies

**Figure 3:**
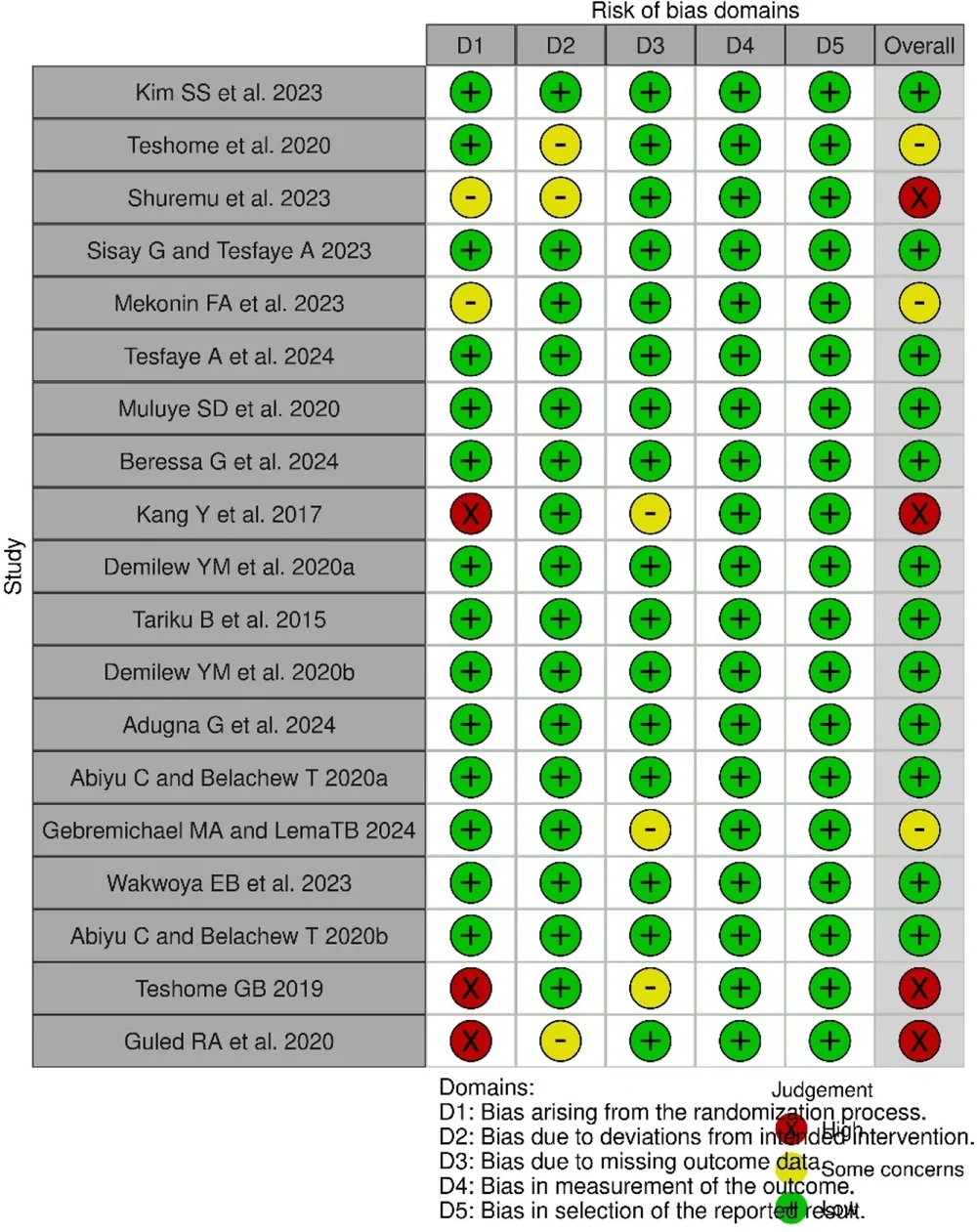
Risk of bias of individual domains of the included RCTs.

**Figure 4:**
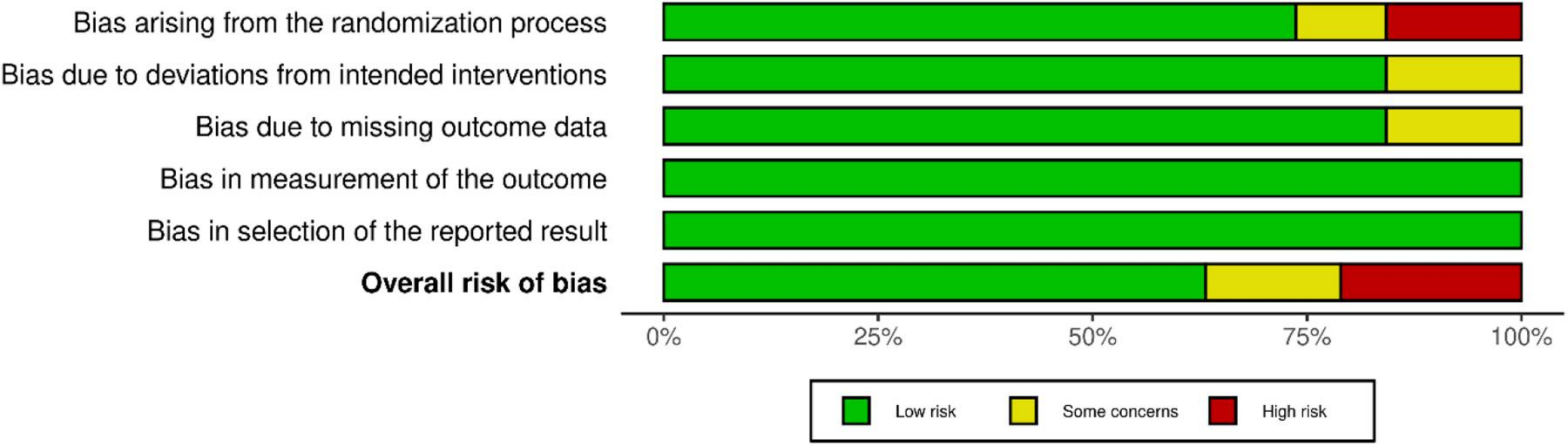
Risk of bias summary, each risk of bias domain presented as percentages across all included studies.

We evaluated 33 studies using RoB 2 for RCTs (19 studies) and ROBINS‐I for non‐randomised studies (14 studies). Overall, the studies showed mixed risk of bias. Among RCTs, concerns varied across the RoB 2 domains, with the most common concerns related to the randomisation process, deviations from intended interventions, and outcome measurement. Overall, 63% of RCTs (*n* = 12/19) had low risk of bias, 16% had some concerns (*n* = 3), and the rest 21% (*n* = 4) had high risk of bias (Figure 3 and Figure 4). On the other hand, 50% of quasi‐experimental studies (*n* = 7) had low risk of bias [detailed report in the supporting material, Additional Tables 3 and 4].

Overall, the majority of studies exhibited low risk of bias in most domains, including deviations from intended interventions, missing outcome data, outcome measurement and selection of reported results. Some concerns were noted primarily in the domains of randomisation and missing outcome data. A high risk of bias was identified in a small proportion of studies for the randomisation process and the overall risk of bias.

i. Effects of SBC on Child Minimum Dietary Diversity (MDD)

**Figure 5:**
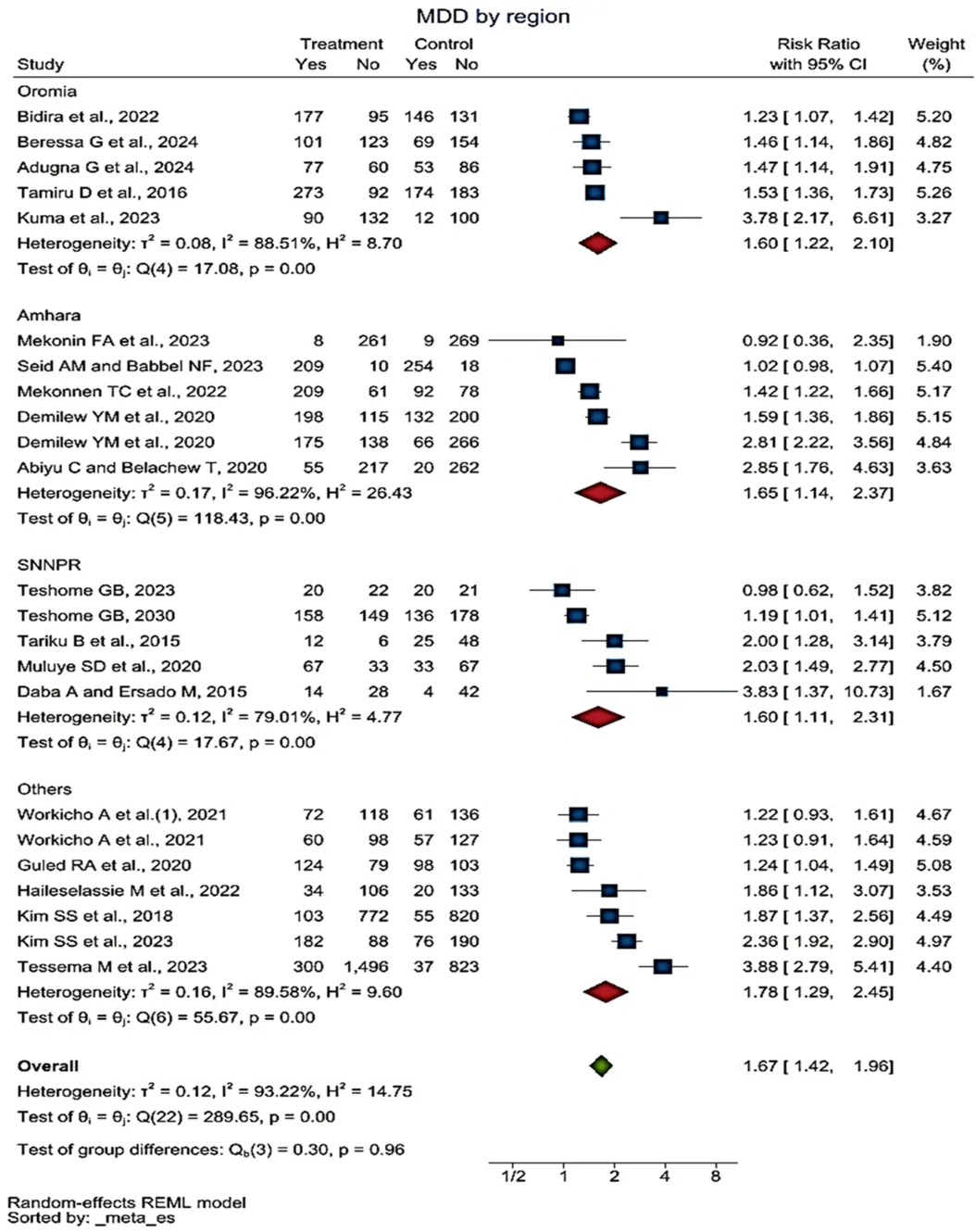
Pooled risk ratios of SBC interventions on achieving Minimum Dietary Diversity (MDD) across Ethiopian regions.

The pooled analysis showed that children whose families or caregivers receiving SBC interventions were 67% more likely to achieve minimum dietary diversity (MDD) compared to those in control groups (RR: 1.67; 95% CI: 1.42–1.96). Regional subgroup analyses revealed consistent positive effects of SBC across all areas studied. The likelihood of meeting MDD increased by 60% in Oromia (RR: 1.60; 95% CI: 1.22–2.10), 65% in Amhara (RR: 1.65; 95% CI: 1.14–2.37), and 60% in SNNPR (RR: 1.60; 95% CI: 1.11–2.31). The strongest effect was observed in other regions, including Addis Ababa, where children were 78% more likely to achieve MDD (RR: 1.78; 95% CI: 1.29–2.45) (Figure 5).

While the main objective of this study is to show regional differences of the effects of the intervention, we analysed other possible variables of interest including design, duration of intervention, MDD cut‐off points, population and risk of bias. In most scenarios, we found that the intervention has significant effects in improving MDD in various contexts. We summarised the synthesis results in the following table (Table 1), forest plots can be accessed from the supporting material, Additional Figure 1. The following subgroup analyses include studies conducted in different population groups and are presented only to explore heterogeneity; the primary meta‐analysis remains restricted to child outcome.

ii. Impact of SBC on Child Dietary Diversity Score (DDS)

**Table 1:** Pooled effects of SBC interventions on achieving MDD, overall and by population and study characteristics.

		Studies (*n*)	RR (95% CI)	*I* ^2^	*p* value
Design	RCT	13	1.75 (1.42 2.15)	79.48	< 0.001
	Quasi	10	1.59 (1.23 2.05)	95.38	< 0.001
Duration	< 6 months	7	1.53 (1.17 1.99)	92.30	< 0.001
	≥ 6 months	16	1.75 (1.42 2.14)	91.13	< 0.001
Classification	≥ 4 food groups	17	1.70 (1.39 2.10)	93.54	< 0.001
	≥ 5 food groups	2	1.41 (1.21 1.64)	0.00	0.37
	Highest tertile	4	1.76 (1.32 2.36)	90.40	< 0.001
Population	Children	13	1.71 (1.38 2.12)	88.52	< 0.001
	Pregnant women	6	1.77 (1.26 2.49)	90.46	< 0.001
	Adolescent girls	3	1.71 (1.12 2.59)	87.39	< 0.001
	Elders	1	1.02 (0.98 1.07)	—	
Quality	High	15	1.88 (1.56 2.27)	89.21	< 0.001
	Moderate	1	0.92 (0.36 2.35)	—	
	Low	7	1.24 (1.07 1.45)	76.00	< 0.001

Abbreviations: CI, confidence interval; RCT, randomised controlled trial; RR, risk ratio.

**Figure 6:**
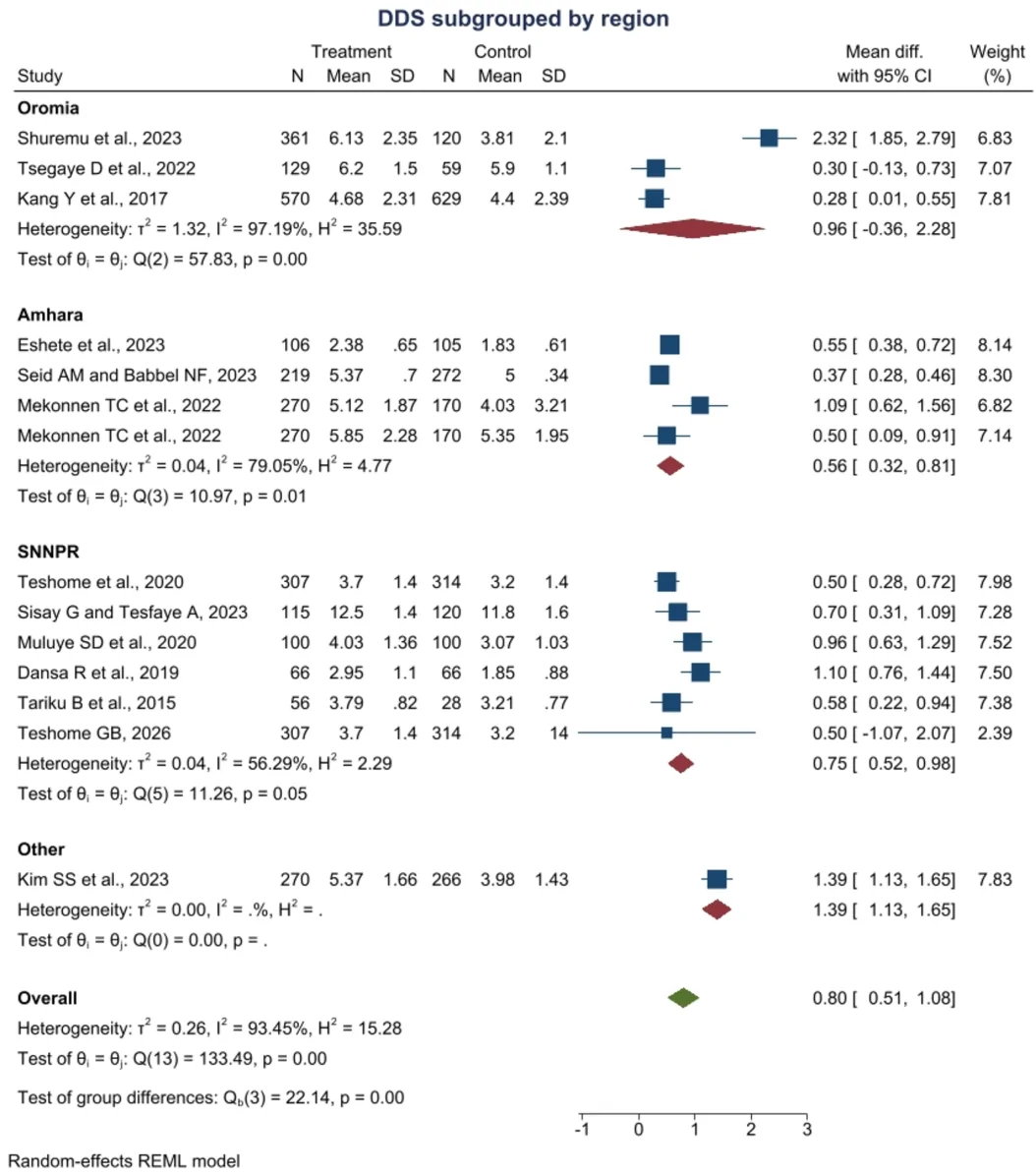
Pooled effects of SBC interventions on Dietary Diversity Score (DDS) by region.

Overall, the pooled analysis indicated that SBC interventions were associated with a significant improvement in DDS, with a pooled mean difference of 0.80 (95% CI: 0.51–1.08). However, the analysis revealed substantial heterogeneity across studies (*I* ^2^ = 94.54%), suggesting differences in intervention design, population characteristics, and implementation contexts. Sub‐group analysis by region highlighted variations in effectiveness. Significant improvements were observed in Amhara (MD: 0.56; 95% CI: 0.32–0.81) and SNNPR (MD: 0.75; 95% CI: 0.52–0.98), while the strongest effect was found in other regions, including Addis Ababa (MD: 1.39; 95% CI: 1.13–1.65). In contrast, the pooled effect in Oromia was positive but not statistically significant (MD: 0.97; 95% CI: –0.36 to 2.29), with extremely high heterogeneity (*I* ^2^ = 98.21%). The significant variation across regions (*p* < 0.001) underscores the importance of tailoring SBC interventions to specific cultural and contextual factors to maximise their impact on dietary diversity, Figure 6.

The subgroup analysis revealed that SBC interventions consistently improved DDS across nearly all categories, though the magnitude of effect varied significantly by study characteristics. Interventions evaluated through RCTs demonstrated a higher mean increase in DDS (MD = 0.92; 95% CI: 0.45, 1.39) compared to quasi‐experimental designs (MD = 0.63). When categorised by target population, the most substantial improvements were observed among adolescent girls (MD = 1.27; 95% CI: 0.98, 1.55), whereas the effect size for children was more modest (MD = 0.62). Notably, high‐quality studies reported a larger pooled effect (MD = 0.92) than moderate or low‐quality studies. Despite these positive trends, high levels of statistical heterogeneity persisted in most subgroups, Table 2. Subgroup forest plots are given in the supporting material, Additional Figure 2.

iii. Impact of SBC on Child Weight Across Regions

**Table 2:** Pooled effects (MD) of SBC interventions on Dietary Diversity Score (DDS) using different subgroups.

		Studies (#)	MD (95% CI)	*I* ^2^	*p* value
Design	RCT	8	0.92 (0.45 1.39)	93.35	< 0.001
	Quasi	6	0.63 (0.36 0.90)	85.30	< 0.001
Food groups	7 groups	10	0.86 (0.47 1.25)	95.91	< 0.001
	8 groups	2	0.78 (0.21 1.36)	70.30	0.07
	Other	2	0.51 (0.12 0.90)	46.15	0.17
Population	Children	7	0.62 (0.39 0.85)	63.01	0.002
	Adolescent girls	2	1.27 (0.98 1.55)	42.93	0.19
	Pregnant women	2	0.51 (0.12 0.90)	46.15	0.17
	Elders	2	1.33 (−0.58 3.24	98.41	< 0.001
	Other	1	0.55 (0.38 0.72)		
Study quality	High quality	4	0.92 (0.56 1.29)	79.19	< 0.001
	Moderate quality	4	0.68 (0.36 1.01)	77.50	0.02
	Low quality	6	0.79 (0.17 1.42)	71.84	< 0.001

Abbreviations: CI, confidence interval; MD, mean difference; RCT, randomised controlled trial.

**Figure 7:**
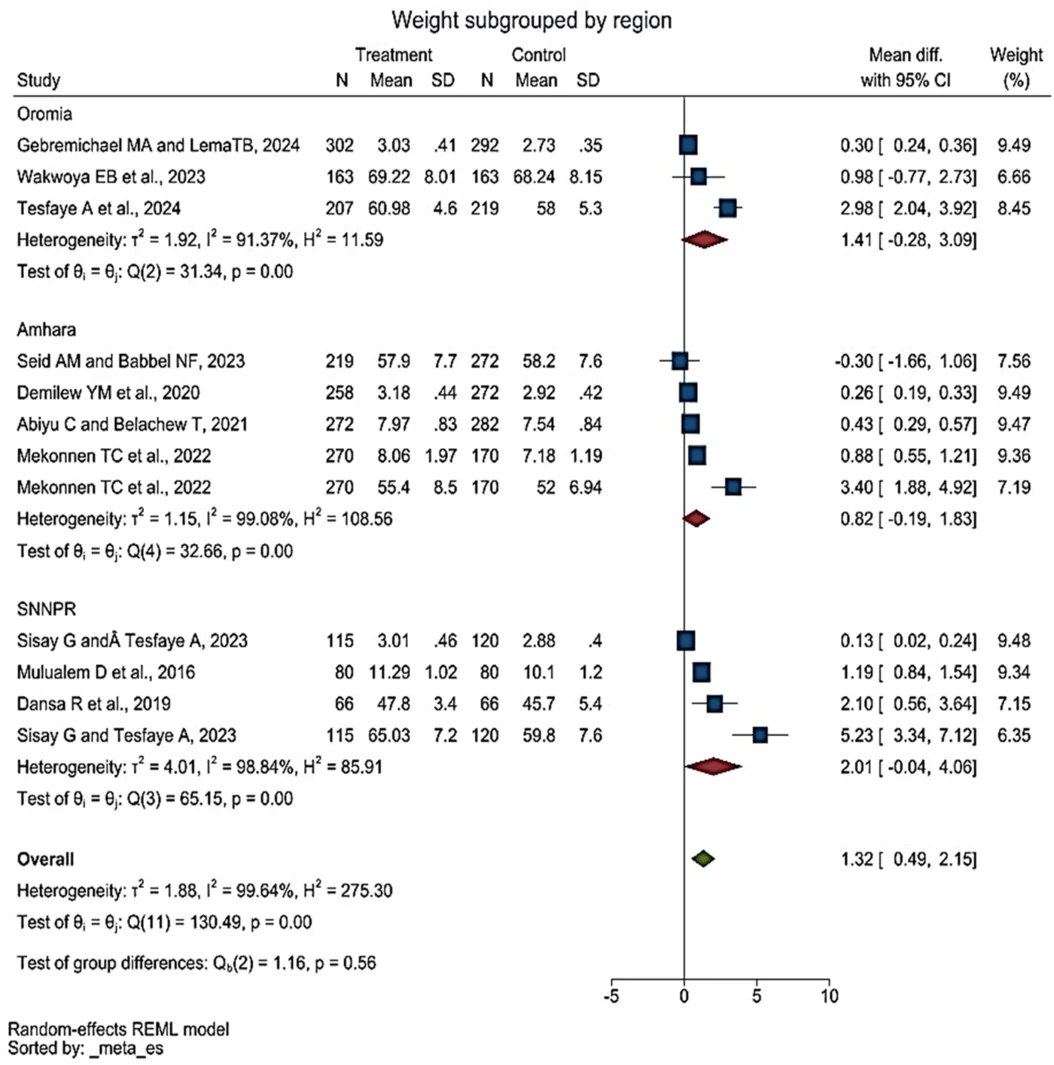
Pooled mean differences in weight following SBC interventions, by study characteristics.

SBC interventions were associated with a significant overall improvement in child weight, with a pooled mean difference of 1.32 (95% CI: 0.49–2.15). Despite this positive effect, heterogeneity across studies was extremely high (*I* ^2^ = 99.64%). At the regional level, the largest improvement was observed in SNNPR (MD: 2.01; 95% CI: –0.04 to 4.06), followed by Oromia (MD: 1.41; 95% CI: –0.28 to 3.09) and Amhara (MD: 0.82; 95% CI: –0.19 to 1.83). However, the effects in Oromia and Amhara were not statistically significant (*p* = 0.56), Figure 7.

The subgroup analysis for child and adult weight outcomes indicates that SBC interventions generally led to a positive increase in mean body weight, though the effect was highly variable across subgroups. When stratified by study design, both RCTs (MD = 1.33; 95% CI: 0.04, 2.62) and quasi‐experimental studies (MD = 1.36; 95% CI: 0.30, 2.43) showed statistically significant weight gains. Notably, the effect size was markedly higher in adult populations (MD = 2.91) compared to children (MD = 0.47), which would be expected due to the absolute weight difference of the two groups. Regarding study quality, high‐quality (MD = 1.54) and moderate‐quality (MD = 0.99) studies reported significant improvements in weight; however, the pooled effect for low‐quality studies was not statistically significant (MD = 1.28; 95% CI: −0.74, 3.30), shown in Table 3 and Additional Figure 3 of the supporting material.

**Table 3:** Pooled mean differences in child weight following SBC interventions sub‐grouped by different variables.

		Studies (#)	MD (95% CI)	*I* ^2^	*p* value
Design	RCT	7	1.33 (0.04 2.62)	99.86	< 0.001
	Quasi	5	1.36 (0.30 2.43)	92.37	< 0.001
Population	Children	7	0.47 (0.17 0.77)	97.34	< 0.001
	Adults	5	2.91 (1.67 4.15)	71.54	0.02
Study quality	High quality	6	1.54 (0.02 3.05)	99.79	< 0.001
	Moderate quality	3	0.99 (0.09 1.88)	94.24	< 0.001
	Low quality	3	1.28 (−0.74 3.30)	90.78	< 0.001

Abbreviations: CI, confidence interval; MD, mean difference; RCT, randomised controlled trial.

### 3.7 Publication Bias and Sensitivity Analysis

Visual inspection of the funnel plots did not suggest substantial asymmetry. However, Egger's regression test indicated statistically significant small‐study effects for MDD (*p* = 0.0458) and weight (*p* = 0.0012), but not DDS (*p* = 0.8925). Trim‐and‐fill analyses imputed no additional studies, suggesting that these small‐study effects did not materially alter the pooled estimates.

## 4 Discussions

Through synthesising available evidence, we demonstrated the substantial impact of SBC interventions on improving child dietary diversity and weight gain outcomes in Ethiopia. Three key outcomes were assessed: Minimum Dietary Diversity (MDD), Dietary Diversity Score (DDS), and child weight gain. Both MDD and DDS are well‐established indicators of dietary diversity and diet quality among young children's diets, and they are sensitive to changes resulting from Behaviour‐focused interventions (World Health Organization & United Nations Children's Fund 2021; Diop et al. 2021; Ngala 2015). Additionally, child weight gain served as a secondary growth indicator, reflecting the cumulative effects of improved feeding practices and overall nutritional status. The findings confirm that SBC strategies, when effectively implemented, can lead to significant improvements not only in dietary diversity but also in child growth outcomes.

Dietary diversity is a fundamental determinant of child nutrition and health, particularly during the first 2 years of life ‐ a period that lays the foundation for optimal growth, brain development, and long‐term well‐being (Saavedra and Dattilo 2022; Mameli et al. 2016). Diets that include a variety of food groups are more likely to meet the micronutrient requirements essential for preventing stunting, wasting, and other forms of malnutrition.

In this review, two primary indicators were examined: MDD and DDS. MDD, a binary measure, assesses whether children consume foods from at least five of the recommended food groups, while DDS provides a continuous score reflecting the variety and quality of foods consumed. Both indicators are widely recognised as sensitive to Behaviour‐focused interventions, including SBC strategies (Diop et al. 2021). By influencing feeding practices, enhancing caregiver knowledge, and shifting social norms, SBC interventions can directly improve these dietary indicators, contributing to better child nutrition outcomes.

Across the 33 studies included in this review, children exposed to SBC interventions were 67% more likely to achieve the MDD compared to those in control groups (RR: 1.67; 95% CI: 1.42–1.96). This strong association underscores the effectiveness of SBC in improving dietary quality. Similarly, the pooled analysis for DDS showed an MD = 0.80; 95% CI: 0.51–1.08, indicating that children in intervention groups consumed, on average, nearly one additional food group compared to controls.

These findings align with global evidence highlighting that SBC interventions—when tailored to local cultural contexts and effectively implemented—can substantially improve child feeding practices (Turk et al. 2025). By enhancing caregiver knowledge, shifting attitudes, and reinforcing positive social norms, SBC strategies not only diversify diets but also lay the groundwork for sustained improvements in child nutrition. Ultimately, such interventions contribute to reducing micronutrient deficiencies (Kennedy et al. 2018; Reerink et al. 2017), promoting healthy growth (Abuye et al. 2024; Turk et al. 2022), and advancing progress toward child survival and development goals.

### 4.1 Effectiveness of SBC on Minimum Dietary Diversity

We found a robust positive association between SBC interventions and the likelihood of children meeting MDD standards. The pooled risk ratio indicates that children exposed to SBC interventions were 67% more likely to consume the minimum number of food groups compared to those in control groups. This finding highlights SBC's critical role in promoting optimal infant and young child feeding (IYCF) practices by enhancing caregiver knowledge, attitudes, and skills.

Subgroup analyses revealed regional variations in effectiveness. Significant improvements were observed in Oromia, Amhara, and SNNPR, with RRs ranging from 1.60 to 1.65, while the strongest effect was reported in other regions, including Addis Ababa (RR: 1.78). Such variability likely reflects differences in intervention fidelity, local food environments, and cultural receptivity to behaviour change messages. Urban areas, where information access and food availability are generally better, may have provided a more enabling environment for SBC to achieve its full impact.

These results are consistent with global evidence from countries such as Bangladesh, Nepal and Malawi, where SBC interventions have similarly increased dietary diversity among young children (Beressa et al. 2024; Turk et al. 2025; Girard et al. 2021). International experiences show that well‐designed SBC programmes—particularly those combining interpersonal counselling with mass media and community engagement—successfully reshape feeding behaviours and social norms. However, global studies also caution that knowledge gains alone may not guarantee improved practices when structural barriers such as poverty, food insecurity and gender inequalities persist (Ruel and Alderman 2013; Gillespie et al. 2015). Therefore, while SBC is a powerful tool for improving IYCF, its impact is potentiated when implemented alongside broader interventions that enhance food access and empower caregivers.

### 4.2 SBC and Dietary Diversity Score Improvements

The analysis further demonstrates that SBC interventions positively influenced the continuous measure of DDS.

Improvements in DDS reinforce the finding that SBC can effectively increase the number of food groups consumed by children, a key determinant of micronutrient adequacy. In practical terms, a 0.8‐point improvement can mean an additional one to two food groups being included in a child's daily diet—a meaningful gain in settings where dietary monotony is prevalent (Arimond and Ruel 2004; Kathryn 2013).

The differential effects across regions may relate to the intensity, duration, and delivery channels of SBC interventions (Workicho et al. 2021; Kim et al. 2016; Turk et al. 2025). For instance, programmes leveraging community health workers, religious leaders, and peer support groups may enjoy deeper community penetration and stronger behaviour change effects. Conversely, regions where SBC was primarily media‐based may face challenges with message retention and behavioural reinforcement. Additionally, the presence of complementary programmes, such as kitchen gardens or food vouchers, may have synergistically enhanced dietary diversity in some contexts.

### 4.3 Regional Patterns of Effectiveness

The effectiveness of SBC on MDD and DDS varied across Ethiopia's diverse sub‐nationals/regions, reflecting contextual differences in food availability, health service access, socio‐cultural norms, and implementation capacity. Oromia, Amhara, and the Southern Nations, Nationalities and Peoples' Region (SNNPR) showed moderate to strong impacts on both MDD and DDS outcomes. In Oromia and SNNPR, the relative likelihood of achieving MDD was 60% higher among children in SBC‐exposed households, while Amhara exhibited a 65% improvement. These gains are likely linked to the relatively well‐developed health systems, extensive health extension networks, and high coverage of community health services in these regions.

The DDS improvements in Amhara (mean difference: 0.56) and SNNPR (0.74) further demonstrate the role of SBC in encouraging the inclusion of diverse food groups. While Oromia showed the highest mean difference in DDS (0.97), the wide confidence interval suggests a degree of variability in effect size, possibly due to inconsistencies in programme delivery or external factors influencing food access.

The most pronounced effects, however, were observed in ‘other’ regions, including Addis Ababa. In these settings, MDD improved by 78% (RR: 1.78) and DDS by 1.39 points (95% CI: 1.13–1.65), the highest among all regions. Urban advantages such as improved health infrastructure, high literacy levels, better access to mass media, and more diverse food markets may amplify the effects of SBC messaging. Urban populations are also more likely to benefit from multiple, reinforcing communication channels, including digital platforms, health facility contacts, and community‐based outreach, all of which contribute to behaviour change.

### 4.4 Constraints in Rural and Underserved Regions

Despite the demonstrated benefits of SBC, rural and pastoralist regions face unique challenges that limit the full realisation of its potential. In these settings, households often encounter environmental constraints such as recurrent droughts, poor soil fertility, and seasonal food shortages that restrict the availability of diverse foods. Economic limitations, including widespread poverty and low market integration, further hinder families' ability to purchase nutrient‐rich items like animal‐source foods, fruits, and vegetables. Thus, even when SBC messages are well understood and positively received, the lack of resources to implement recommended feeding practices undermines their effectiveness.

In Oromia, for example, while improvements in DDS were observed, the non‐significant results suggest that structural barriers—such as uneven implementation coverage, inadequate programme intensity, and poor supply chains—may have diluted the intervention's impact (Victora et al. 2016; Bhutta et al. 2013). Rural health systems often face shortages of trained health workers, weak supervisory mechanisms, and logistical difficulties in reaching remote households. Moreover, pastoralist and nomadic communities present distinct challenges for SBC programming due to their high mobility, reliance on livestock‐based diets, and cultural practices that may not align with standard nutrition messages. Addressing these constraints requires adapting SBC approaches to local realities and complementing them with interventions that strengthen food systems, improve service delivery, and enhance resilience to environmental shocks.

### 4.5 Determinants of Regional Variation

The varying effectiveness of SBC interventions across Ethiopia's regions can be attributed to a combination of contextual and structural factors that shape how messages are delivered, received, and translated into behaviour change.

One of the most influential factors is the food environment and household food security (Kim et al. 2016). In regions where diverse foods are accessible and affordable, caregivers are better positioned to act on SBC messages encouraging varied diets for children. However, in food‐insecure areas—where availability is constrained by environmental challenges or market access— even the most persuasive SBC efforts may fall short. Without tangible improvements in food availability, families may be unable to implement recommended practices despite understanding their benefits.

Another key determinant is the strength of the local health system. Regions with well‐staffed health centres and trained personnel tend to deliver SBC interventions more consistently and effectively. Where community health workers receive regular supervision and training, and where they are trusted by the populations they serve, SBC programmes are more likely to gain traction. These regions benefit from a reliable workforce that reinforces health messages through repeated and personalised engagement.

Sociocultural norms also play a significant role in shaping dietary behaviours and the receptiveness to SBC. In communities where traditional beliefs influence child feeding practices—such as restricting animal‐source foods for young children or limiting women's dietary intake—SBC must be culturally sensitive. Messages delivered through respected community figures, like elders or religious leaders, often resonate more deeply and facilitate greater behaviour change in such settings.

The type and accessibility of communication platforms further influence SBC effectiveness. In urban areas and regions with higher literacy rates, platforms such as radio, television, and mobile messaging can reach wide audiences efficiently (Noar et al. 2011). However, in rural or remote areas with limited infrastructure, face‐to‐face communication methods— such as home visits, community theatre, or pictorial materials— tend to be more impactful. The choice of platform must align with both the audience's accessibility and preferences.

Lastly, the degree to which SBC is integrated with other health and nutrition services affects its success. When SBC is combined with interventions like kitchen gardening, school meal programmes, or cash transfers, households are more likely to have the means and motivation to adopt new behaviours. This integration creates a supportive environment where knowledge is reinforced by tangible resources and community‐wide engagement, making sustained change more feasible.

Together, these interrelated factors highlight the complexity of delivering SBC at scale in a diverse country like Ethiopia. Understanding and addressing these regional determinants is essential to ensuring that SBC interventions are not only effective but also equitable (Avula et al. 2017).

These regional comparisons should be interpreted cautiously. The subgroup analyses compared studies conducted in different regions rather than identical interventions implemented across regions. Consequently, observed regional differences may reflect variation in intervention design, implementation fidelity, duration, delivery platforms, target populations or study quality rather than intrinsic regional differences. Because these factors were not fully controlled, the regional findings should be regarded as hypothesis‐generating rather than definitive evidence of regional effectiveness.

### 4.6 Implementation Gaps and Opportunities

While the review confirms SBC's value, it also highlights several implementation gaps. First, not all interventions reported detailed descriptions of SBC components, delivery frequency, or audience segmentation—factors essential for replication and scale‐up. Second, few studies systematically evaluated message comprehension or behavioural drivers across target groups, limiting insights into what works for whom and why.

A key opportunity lies in leveraging Ethiopia's Health Extension Programme as a platform for localised, equity‐focused SBC. However, this requires consistent investment in training, job aids, supervision, and motivational incentives for frontline workers. Moreover, integrating nutrition messaging into broader community development programmes—especially in regions facing high stunting or wasting rates—can create synergistic effects.

Digital innovation also offers promise. Mobile phone‐based messaging and voice‐based interventions may provide scalable channels for delivering nutrition information and behaviour‐change support to caregivers, including populations with limited access to conventional health services (Thepha et al. 2026; Downs et al. 2019). However, digital SBC must be carefully designed to avoid excluding the poorest or most remote households.

### 4.7 Policy Implications

The regional disparities in SBC effectiveness underline the need for decentralised programme design and implementation. National‐level guidelines should serve as a framework, but regions must have the capacity to adapt SBC strategies to their specific contexts. This includes translating materials into local languages, involving regional cultural leaders, and modifying communication channels based on infrastructure and audience preference.

Additionally, monitoring and evaluation systems should disaggregate SBC outcomes by region, gender and socio‐economic status. This will help identify lagging areas, assess intervention equity and inform responsive adjustments. Creating feedback mechanisms at the community level can also ensure that interventions remain relevant and trusted over time.

### 4.8 Strengths and Limitations of the Review

This review is the first of its kind to synthesise sub‐national evidence on SBC nutrition outcomes across Ethiopia. Its strengths include a comprehensive search strategy, adherence to PRISMA guidelines and inclusion of diverse study designs. It provides a nuanced understanding of where and how SBC works, and where it needs strengthening.

However, several limitations must be acknowledged. First, the quality of included studies varied, with some lacking rigorous design or complete reporting. Second, the high heterogeneity limits the precision of pooled estimates, especially for weight outcomes. Third, the review relied on published literature and only accessible grey literature which might miss some relevant grey literature or unpublished programme reports, which could bias findings. Considerable heterogeneity in intervention content, duration, delivery platforms, implementation fidelity, and target populations also limits causal interpretation of subgroup differences.

Additionally, the temporal scope of studies varied widely, with some conducted during periods of major health system disruption, such as the COVID‐19 pandemic or regional conflict. These contextual factors may have influenced implementation quality and outcome measurement.

Our review indicates that SBC interventions can improve child nutrition outcomes in Ethiopia, particularly dietary diversity. However, effects varied substantially across regions and residential settings. The largest pooled effects were observed in some urban settings, particularly Addis Ababa, whereas effects were more variable in agrarian regions such as Oromia and Amhara. This variation may reflect differences in infrastructure, health‐service capacity, intervention delivery and cultural contexts across settings.

To achieve equitable national impact, a one‐size‐fits‐all model may be insufficient. This requires building local implementation capacity, investing in context‐appropriate communication channels like community radio, and establishing robust regional monitoring systems. By systematically addressing these contextual barriers, Ethiopia can enhance the equity and overall effectiveness of its nutrition interventions. Although regional subgroup analyses suggest context‐specific differences in intervention effectiveness, these findings should be interpreted cautiously because intervention characteristics also differed across studies. Future implementation research directly comparing standardised SBC interventions across regions would help distinguish contextual effects from differences in intervention design and delivery.

Future research should prioritise underrepresented pastoralist and conflict‐affected regions, employ standardised metrics, and investigate long‐term impacts, cost‐effectiveness, and the influence of gender dynamics to better guide effective SBC interventions.

## Author Contributions

T.A.Z., A.S., M.G., A.A.T., and T.G. conceived the study. T.A.Z. and A.A.T. developed the analytical framework, conducted the analysis, and drafted the manuscript. B.T., F.Z., M.B., and A.S. contributed to the conceptualisation and methodological development and provided critical scientific oversight. T.A.Z., A.A.T., A.S., and M.T. contributed to data curation, analysis, interpretation, and visualisation. All authors contributed to interpretation of the findings, critically reviewed and revised the manuscript, approved the final version, and agreed to be accountable for the work.

## Conflicts of Interest

The authors declare no conflicts of interest.

## Policy on Using ChatGPT and Similar AI Tools

During preparation of this manuscript, the corresponding author used ChatGPT (GPT‐4) solely for language polishing and identification of typographical errors. All outputs were reviewed and approved by the authors, who take full responsibility for the manuscript. No AI was used for scientific content, data extraction, study selection, statistical analysis, or reference generation. No AI system is listed as an author or cited as a source.

## Supporting information


**Figure S1:** Subgroups of MDD.
**Figure S2:** DDS subgroups.
**Figure S3:** Weight subgroups.
**Figure S4:** Publication bias.
**Figure S5:** Sensitivity analysis.
**Table S1:** Search strategy.
**Table S2:** Characteristics of included studies.
**Table S3:** Risk of bias of included RCTs.
**Table S4:** Risk of bias for quasi‐experimental studies.

## Data Availability

Data sharing not applicable to this article as no datasets were generated or analysed during the current study. No new datasets were generated. Data analysed in this systematic review and meta‐analysis were extracted from the studies included in the review and are reported in the manuscript and supporting materials.
