# Supplementary material for: Effectiveness of Context‐Specific Social and Behaviour Change (SBC) Interventions in Improving Child Diet in Ethiopia: A Systematic Review and Meta‐Analysis
Source: Matern Child Nutr. 2026 Sep 6;22(3):e70245. doi: 10.1111/mcn.70245 (PMC13546740; doi:10.1111/mcn.70245)
Supplement: Supplementary file 1 [file MCN-22-e70245-s001.docx]

**Effectiveness of Context-Specific Social and Behavioral Change (SBC) Interventions in Improving Child Diet in Ethiopia: *A Systematic Review and Meta-Analysis.***

***Supplementary material***

# Additional Table 1: Search strategy

Query

((((((("Nutrition education") OR ("Nutrition intervention")) OR ("Nutrition promotion")) OR ("Nutrition literacy")) OR ("Nutrition counselling")) AND ((((((((((((nutri*) OR (diet*)) OR (food*)) OR (vitamin*)) OR (nutritional status[MeSH Terms])) OR (nutritive value[MeSH Terms])) OR (healthy, eating[MeSH Terms])) OR (diet[MeSH Terms])) OR (infant nutrition disorders[MeSH Terms])) OR (nutritional requirements, malnutrition[MeSH Terms])) OR (diet, food, and nutrition[MeSH Terms])) OR (feed*))) AND (Ethiopia[Text Word])) NOT ((("Systematic review"[Title]) OR ("Meta analysis"[Title])) OR ("Scoping review"[Title]))

(("Systematic review"[Title]) OR ("Meta analysis"[Title])) OR ("Scoping review"[Title])

"Scoping review"[Title]

"Meta analysis"[Title]

"Systematic review"[Title]

(((((("Nutrition education") OR ("Nutrition intervention")) OR ("Nutrition promotion")) OR ("Nutrition literacy")) OR ("Nutrition counselling")) AND ((((((((((((nutri*) OR (diet*)) OR (food*)) OR (vitamin*)) OR (nutritional status[MeSH Terms])) OR (nutritive value[MeSH Terms])) OR (healthy, eating[MeSH Terms])) OR (diet[MeSH Terms])) OR (infant nutrition disorders[MeSH Terms])) OR (nutritional requirements, malnutrition[MeSH Terms])) OR (diet, food, and nutrition[MeSH Terms])) OR (feed*))) AND (Ethiopia[Text Word])

Ethiopia[Text Word]

(((((((((((nutri*) OR (diet*)) OR (food*)) OR (vitamin*)) OR (nutritional status[MeSH Terms])) OR (nutritive value[MeSH Terms])) OR (healthy, eating[MeSH Terms])) OR (diet[MeSH Terms])) OR (infant nutrition disorders[MeSH Terms])) OR (nutritional requirements, malnutrition[MeSH Terms])) OR (diet, food, and nutrition[MeSH Terms])) OR (feed*)

diet, food, and nutrition[MeSH Terms]

nutritional requirements, malnutrition[MeSH Terms]

infant nutrition disorders[MeSH Terms]

diet[MeSH Terms]

healthy, eating[MeSH Terms]

nutritive value[MeSH Terms]

nutritional status[MeSH Terms]

vitamin*

food*

feed*

diet*

nutri*

(((("Nutrition education") OR ("Nutrition intervention")) OR ("Nutrition promotion")) OR ("Nutrition literacy")) OR ("Nutrition counselling")

"Nutrition counselling"

"Nutrition literacy"

"Nutrition promotion"

"Nutrition intervention"

"Nutrition education"

# Additional Table 2: Characteristics of included studies

|  | Author | Year | Region | Design | Population | Age (Range/Mean) | Sample-I | Sample-C | Intervention areas (topics) | Intervention context | Mode of intervention | Duration of intervention | Outcomes |
| --- | --- | --- | --- | --- | --- | --- | --- | --- | --- | --- | --- | --- | --- |
| 1 | Kim SS et al. | 2023 | SNNPR and Somali | RCT | Adolescent girls | 10-14 | 270 | 266 | dietary diversity, energy adequacy, and healthy food choices | School | flag ceremonies, classroom lessons, school club meetings, peer group mentoring, counselling, and parent–teacher meetings | 4 months | DDS, MDD |
| 2 | Teshome et al. | 2020 | Sidama | RCT | Mother child pairs | 25.5 | 307 | 314 | balanced diet, food groups and importance of pulses, food processing | HI | Training, counselling, posters, demonstrations, recipe preparations | 9 months | DDS |
| 3 | Eshete et al. | 2023 | Amhara | QE | Adults | 48-63 | 106 | 105 | Balanced diet, healthy eating habits, how to prepare and cook healthy food | HI | lecture, group discussion and individual consultation | 6 months | DDS |
| 4 | Shuremu et al. | 2023 | Oromia | RCT | Elders | >60 | 361 | 359 | Healthy eating habits, life style changes, behavioral risk factors, physical exercise | Community based | Brainstorming Role play, group discussion Illustrative and interactive lecture | 6 months | DDS |
| 5 | Kuma et al. | 2023 | Oromia | RCT | Pregnant women | 23.77 | 224 | 112 | Nutrition education and birth preparedness, complication readiness | HI and home visit | Direct information provision, sharing experiences, Discussions, Demonstrations, Telling stories, leaflets with the key messages | 6 months | MDD |
| 6 | Sisay G and Tesfaye A | 2023 | SNNPR | RCT | Pregnant women | 26 | 115 | 120 | nutritional value of food, balanced diet, cooking practices for optimum retention of nutrients, food taboos, health and hygiene practices, disease control, food frequency | HI | Lectures, Q&A role play, drama, brochures, leaflets photograph and counselling | throughout pregnancy | DDS,  Weight |
| 7 | Mekonin FA et al. | 2023 | Amhara | RCT | Adolescent girls | 15-19 | 269 | 278 | pulses-based nutrition education | School based | lecture and group discussion with the aid of flip charts, posters and leaflets | 4 weeks | MDD |
| 8 | Bidira et al. | 2022 | Oromia | QE | Preschool children | 2--5 | 272 | 277 | healthy diet awareness, nutrition and hygiene, child feeding | School, HI | Group discussions, lectures, role‐plays, active participation and demonstration | 5 months | MDD |
| 9 | Tsegaye D et al. | 2022 | Oromia | QE | Pregnant women |  | 129 | 117 | the role of nutrients in promoting good health, the consequences of inadequate nutrient intake, key recommendations, adherence to the iron/folic acid | HP and home to home visit | Direct (teacher‐directed) and interactive (discussion, sharing) | 3 months | DDS |
| 10 | Seid AM and Babbel NF | 2023 | Amhara | QE | Elders | >60 | 219 | 272 | balanced diet intake; meal frequency, food varieties, consumption of health diet | Home to home | face-to-face counselling, flyer | 1 month | Weight, MDD, DDS |
| 11 | Haileselassie M et al. | 2022 | Tigray | QE | Children 6-23 months | 6-23 months | 140 | 153 | child undernutrition, why nutrition matters during the first two years of life, feeding principles and practices, use of ASFs for children, feeding frequency, and food groups | Home to home | counselling resources (such as pictures and leaflets) | 9 months | MDD |
| 12 | Tesfaye A et al. | 2024 | Oromia | RCT | Pregnant adolescents | 15-19 | 207 | 219 | Diversified diet, increasing the meal frequency and portion size as gestational age increased | Community based | GALIDRAA approach (Greet, Ask, Listen, Identify, Discuss, Repeat, Agree, and Appoint) | 2 months | weight |
| 13 | Muluye SD et al. | 2020 | SNNPR | RCT | Children 6-23 months | 6-23 months | 100 | 100 | complementary food recipes and preparation, appropriate amount and frequency of feeding, personal hygiene and sanitation, and dietary diversification | daycare center | Active lecturer, posters, note pad, brochures, and practical demonstration session | 4 months | DDS, MDD |
| 14 | Mekonnen TC et al. | 2022 | Amhara | QE | Mother child pairs (<12 months) |  | 270 | 170 | diversified food production via organic agriculture, modification, complementary food preparation, and feeding behavior | Community based and home visit | sensitization workshops, meetings, food festivals, and experience-sharing through the community-field visits | 1 year | DDS, weight,  MDD |
| 15 | Beressa G et al. | 2024 | Oromia | RCT | Pregnant women |  | 224 | 223 | nutrition education, counseling cards, and a structured work schedule | HI and home | Presentation, discussion, demonstration, and picture-based activities were used to deliver counselling | 6 months | MDD |
| 16 | Kang Y et al. | 2017 | Oromia | RCT | Undernourished children | 6-24 month | 570 | 629 | breast-feeding or complementary feeding | At kebele | Discussions, demonstrations | 2 weeks | DDS |
| 17 | Dansa R et al. | 2019 | SNNPR | QE | Adolescent girls | 10-19 | 66 | 66 | food groups and diet diversity, nutritional benefits of pulses, household pulse preparation and processing techniques, pulse-incorporated food preparation, and consumption, proper food hand-ling and storage | School based | lesson-based education, group discussion, and demonstrations of pulse recipe preparation skills | 6 months | DDS, Weight |
| 18 | Demilew YM et al. | 2020 | Amhara | RCT | Pregnant women |  | 313 | 332 | increasing meal frequency and portion size with increasing gestational age | Home to home | client-centered approach to identify women’s dietary practices and their specific needs | throughout pregnancy | MDD, |
| 19 | Tamiru D et al. | 2016 | Oromia | QE | primary school Students | 10-19 | 365 | 357 | developing the young children’s knowledge, attitudes, beliefs, and skills to enable them to be responsible for and to protect their own health | School based | peer-led approach and health promotion through school media and health clubs, and community and school events, role-playing, posters, debates, drama and audio-visual activities | 8 months | MDD |
| 20 | Mulualem D et al. | 2016 | SNNPR | QE | Children | 6-18 months | 80 | 80 | complementary feeding practices | Near home | education lessons, group discussions, recipe-based demonstration, | 6 months | weight |
| 21 | Tariku B et al. | 2015 | SNNPR | RCT | mother child pairs | 6-18 month | 56 | 56 | Complementary feeding | Home visits and site | personalized active discussions | 6 months | DDS, MDD |
| 22 | Demilew YM et al. | 2020 | Amhara | RCT | Pregnant women |  | 258 | 272 | increasing meal frequency and portion size with increasing gestational age | Home to home | client-centered approach to identify women’s dietary practices and their specific needs | throughout pregnancy | weight |
| 23 | Adugna G et al. | 2024 | Oromia | RCT | Mother child pairs | 6-23 month | 137 | 139 | complementary feeding, the importance of breastfeeding, knowledge of dietary diversity, and dietary diversity practice |  | oral presentation and poster | 3 months | MDD |
| 24 | Abiyu C and Belachew T | 2020 | Amhara | RCT | Mother child pairs | 6-23 months | 272 | 282 | Complementary feeding | Home visits and group trainings | Talks, group discussions, group work exercises, demonstrations, role plays, storytelling, simulation, case studies and problem-solving, cooking demonstrations, and feedback | 9 months | MDD |
| 25 | Abiyu C and Belachew T | 2021 | Amhara | RCT | Mother child pairs | 6-23 months | 272 | 282 | Complementary feeding | Home visits and group trainings | Talks, group discussions, group work exercises, demonstrations, role plays, storytelling, simulation, case studies and problem-solving, cooking demonstrations, and feedback | 9 months | Weight |
| 26 | Gebremichael MA and LemaTB | 2024 | Oromia | RCT | Pregnant women | 18-49 | 302 | 292 | Dietary diversity, meal frequency, portion size, iodized salt, INT utilization, personal hygiene, rest, health service utilization | Site training and home visits | role-playing, group meal preparation, individualized counseling and support during each home visit | 4 months | weight |
| 27 | Wakwoya EB et al. | 2023 | Oromia | RCT | Pregnant women | 18-45 | 163 | 163 | Healthy eating habit, diet diversification, adequate energy intake, iron and folic acid, iodized salt, rest, deworming, ITN and health service use | HI | presentation, discussions, role play, SMS, brochures | Trimester based | Weight |
| 28 | Tessema M et al. | 2023 | Amhara, Oromia, SNNPR | QE | Under 2 years old | <2 | 1796 | 860 | Dietary diversity, child feeding and nutrition sensitive agriculture |  | Couple counselling, women-men dialogue, demonstration, media message |  | MDD |
| 29 | Workicho A et al. | 2021 | Amhara and Oromia | QE | Pregnant or lactating women |  | 158 | 184 | health service utilization, antenatal care, nutrition counselling, initiation of breastfeeding, exclusive breastfeeding, complementary feeding, dietary diversity, role of husbands in childcare and nutrition, women empowerment in household decision making, and nutrition communication | Home | Listening of pre-recorded audio material | 10 months | MDD |
| 30 | Kim SS et al. | 2016 | Tigray and SNNPR | QE | Mother child pairs | 0-59 months | 619 | 606 | infant and young children feeding practice | Home and HI | Radio messages, cooking demonstration, print material | 6 months | MDD |
| 31 | Teshome GB | 2019 | SNNPR | RCT | Mother child pairs | 6-24 months | 42 | 41 | diet diversification; the benefits of pulses; household pulse processing and preparation techniques, and the need to prepare and cook a variety of pulse-based dishes, including pulse-cereal mix complementary food | Site training and home visits | Training, counselling, posters, demonstrations, recipe preparations | 9 months | MDD |
| 32 | Guled RA et al. | 2020 | Somali | RCT | Mother child pairs | 6-59 months | 203 | 201 | nutrition for pregnant mother, delivery, correct breastfeeding, positioning and attachment, feeding of low birthweight, complementary feeding, and specific health services like; vaccination, deworming, vitamin A, ITNs usage, and environmental sanitation | HI | presentation, roleplay, and demonstrations, posters | 8 months | MDD, |
| 33 | Daba A & Ersado M | 2015 | SNNPR | QE | Children | 6-24 months | 42 | 46 | optimal breast feeding, verity/diet diversity, feeding frequency, food consistency, feeding amount, food hygiene, feeding sick child and responsive feeding | Kebele | Poster, pictures, demonstration and practical sessions | 6 months | MDD |

The table indicted the characteristics of included studies. Year indicates publication year. The design of the studies was either randomized control trial (RCT) or quasi-experimental (QE). The population of the studies were mainly mother child pairs, where, there was intervention on mothers and evaluation of child nutrition outcomes. The age of participants was reported in the form of ranges or mean, unless specified, age is provided in years. We also provided the sample size for intervention group (Sample-I) and control group (Sample-C). Intervention indicates the specific areas (topics) of nutrition education coverage. Intervention could be conducted in schools, health institutions (HI) including health center and health posts, schools, community based at kebele or other specific sites, or home to home – which represents context. Modes of intervention (health education) encompasses the delivery strategies and tools used during the intervention. The duration indicates the span of the intervention. In the outcome column, main outcomes extracted for this study were listed. DDS-dietary diversity score, MDD-minimum dietary diversity, SNNPR-South Nations Nationalities and People’s Region.

Risk of Bias results

# Additional Table 3: Risk of bias of included RCTs

| **Author** | **D1** | **D2** | **D3** | **D4** | **D5** | **Overall** |
| --- | --- | --- | --- | --- | --- | --- |
| Kim SS et al. 2023 | Low | Low | Low | Low | Low | Low |
| Teshome et al. 2020 | Low | Some Concern | Low | Low | Low | Some Concern |
| Shuremu et al. 2023 | Some Concern | Some Concern | Low | Low | Low | High |
| Sisay G and Tesfaye A 2023 | Low | Low | Low | Low | Low | Low |
| Mekonin FA et al. 2023 | Some Concern | Low | Low | Low | Low | Some Concern |
| Tesfaye A et al. 2024 | Low | Low | Low | Low | Low | Low |
| Muluye SD et al. 2020 | Low | Low | Low | Low | Low | Low |
| Beressa G et al. 2024 | Low | Low | Low | Low | Low | Low |
| Kang Y et al. 2017 | High | Low | Some Concern | Low | Low | High |
| Demilew YM et al. 2020a | Low | Low | Low | Low | Low | Low |
| Tariku B et al. 2015 | Low | Low | Low | Low | Low | Low |
| Demilew YM et al. 2020b | Low | Low | Low | Low | Low | Low |
| Adugna G et al. 2024 | Low | Low | Low | Low | Low | Low |
| Abiyu C and Belachew T 2020a | Low | Low | Low | Low | Low | Low |
| Gebremichael MA and LemaTB 2024 | Low | Low | Some Concern | Low | Low | Some Concern |
| Wakwoya EB et al. 2023 | Low | Low | Low | Low | Low | Low |
| Abiyu C and Belachew T 2020b | Low | Low | Low | Low | Low | Low |
| Teshome GB 2019 | High | Low | Some Concern | Low | Low | High |
| Guled RA et al. 2020 | High | Some Concern | Low | Low | Low | High |

D1: Bias arising from the randomization process, D2: Bias due to deviations from intended interventions, D3: Bias due to missing outcome data, D4: Bias in measurement of the outcome, D5: Bias in selection of the reported result

# Additional Table 4: Risk of bias for quasi-experimental studies

| **Authors** | **D1** | **D2** | **D3** | **D4** | **D5** | **D6** | **D7** | **Overall** |
| --- | --- | --- | --- | --- | --- | --- | --- | --- |
| Eshete et al. 2023 | Some Concern | Low | Low | Low | Low | Low | Low | Some Concern |
| Bidira et al. 2022 | Low | Low | Low | Low | Low | Low | Low | Low |
| Tsegaye D et al. 2022 | Low | Low | Low | Low | Low | Low | Low | Low |
| Seid AM and Babbel NF 2023 | Some Concern | Low | Low | Low | Some Concern | Low | Low | High |
| Haileselassie M et al. 2022 | Some Concern | Low | Some Concern | Low | Low | Some Concern | Low | High |
| Mekonnen TC et al. 2022 | Some Concern | High | Low | Low | Low | Low | Low | High |
| Dansa R et al. 2019 | Some Concern | Low | Low | Low | Low | Low | Low | Some Concern |
| Tamiru D et al. 2016a | Low | Low | Low | Low | Low | Low | Low | Low |
| Tamiru D et al. 2016b | Low | Low | Low | Low | Low | Low | Low | Low |
| Mulualem D et al. 2016 | Some Concern | Low | Low | Low | Low | Low | Low | Some Concern |
| Tessema M et al. 2023 | Low | Low | Low | Low | Low | Low | Low | Low |
| Workicho A et al. 2021 | Low | Low | Low | Low | Low | Low | Low | Low |
| Kim SS et al. 2016 | Low | Low | Low | Low | Low | Low | Low | Low |
| Daba A and Ersado M 2015 | Some Concern | Low | Some Concern | Low | Low | Some Concern | Low | High |

D1: Bias due to confounding, D2: Bias in selection of participants into the study, D3: Bias in classification of interventions, D4: Bias due to deviations from intended interventions, D5: Bias due to missing data, D6: Bias in measurement of outcomes, D7: Bias in selection of the reported result

Subgroup analysis results

# Additional Figure 1: Subgroups of MDD

# Additional Figure 2: DDS subgroups

# Additional Figure 3: Weight subgroups

# Additional Figure 4: Publication bias

# Additional Figure 5: Sensitivity analysis
